# Antibiotic Minocycline Prevents Respiratory Syncytial Virus Infection

**DOI:** 10.3390/v11080739

**Published:** 2019-08-11

**Authors:** Swapnil S. Bawage, Pooja M. Tiwari, Shreekumar Pillai, Vida A. Dennis, Shree R. Singh

**Affiliations:** Center for NanoBiotechnology Research, Life Science Building, Harris way, Montgomery, AL 36104, USA

**Keywords:** pneumonia, bronchiolitis, antiviral, antibiotics, minocycline, RSV, Parkin-Ubiquitin pathway, respiratory diseases, neurodegenerative diseases

## Abstract

Treatment drugs, besides their specific activity, often have multiple effects on the body. The undesired effect of the drug may be repurposed as therapeutics, saving significant investigative time and effort. Minocycline has anti-cancer, anti-oxidant, anti-inflammatory, and anti-apoptotic properties. Presently, minocycline is also known to show anti-viral activity against Influenza virus, Japanese encephalitis virus, Simian immunodeficiency virus, Human immunodeficiency virus and West Nile virus. Here, we investigate the effect of minocycline on Respiratory syncytial virus (RSV), a common respiratory virus that causes severe mortality and morbidity in infants, children, and older adult populations. Currently, there is no effective vaccine or treatment for RSV infection; hence, there is a critical need for alternative and effective drug choices. Our study shows that minocycline reduces the RSV-mediated cytopathic effect and prevents RSV infection. This is the first study demonstrating the anti-viral activity of minocycline against RSV.

## 1. Introduction

Worldwide, Respiratory syncytial virus (RSV) is one of the most common causes of lower respiratory tract infections among pediatric and geriatric populations, thereby imposing a hefty healthcare and economic burden [1]. In the United States, the average annual hospitalizations of children younger than one year old or five years old are 100,000–126,000 and 57,527, respectively. Adults older than 65 years have average hospitalization rates of 177,000/year [2]. In 2005, it was estimated that RSV-associated acute lower respiratory infections resulted in 66,000–199,000 deaths in children younger than five years [3]. Currently, the only Food and Drug Administration approved antiviral therapy for this negative sense ssRNA virus is ribavirin and palivizumab (a monoclonal antibody). It is noted that ribavirin faces criticisms for its efficacy and side-effects [4], while palivizumab is expensive and administered as prophylaxis only for high risk patients [5]. Thus, there is an urgent need for alternative drugs to counter RSV.

Multiple approaches of RSV prevention (DNA vaccines, subunit vaccines, and nanovaccines) and treatment (fusion inhibitors, RNAi, and nanoparticles) are still under development or in clinical trials [6]. To manifest as an approved effective drug from a developed drug, a candidate requires decades of rigorous screening and trials [7]. It is sometimes easier to research alternative usage of an already approved drug. One such drug is minocycline; a tetracycline-class broad-spectrum antibiotic, used originally to treat various bacterial infections, that is now also used for treating neurological disorders. Minocycline is known to exhibit antioxidant, anti-inflammatory, antiapoptotic, and anti-cancer properties [8], which makes it desirable for the treatment of multiple sclerosis as well as Alzheimer’s, Huntington’s, and Parkinson’s diseases. Additionally, minocycline has been shown to be an effective antiviral drug against Japanese encephalitis virus (JEV) [9], Simian immunodeficiency virus (SIV) [10], Human immunodeficiency virus (HIV) [11], West Nile virus (WNV) [12], and Influenza virus [13]. These considerations instigated us to investigate the anti-viral activity of minocycline against RSV. We show a distinct effect of minocycline on both RSV infected and uninfected cells with respect to cell morphology and cytokine production. To our knowledge, this is the first study demonstrating the anti-viral activity of minocycline against RSV.

## 2. Materials and Methods

### 2.1. Cell Culture

Human epithelial type-2 cells (HEp-2, CCL-23) and RSV long strain (VR-26), were procured from American Type Culture Collection (ATCC, Manassas, VA, USA). HEp-2 cells were grown in minimum essential medium (MEM) with 10% fetal bovine serum (FBS), L-glutamine (2 mM), PKS (penicillin (75 U/mL), kanamycin (100 μg/mL) and streptomycin (75 μg/mL)) designated as MEM-10. Cells were provided with 2% FBS in MEM whenever RSV infection was performed. Fresh minocycline stock (Sigma, St. Louis, MO, USA) was made in sterile distilled water for each experiment and diluted to desired concentration in MEM.

### 2.2. Cell Viability Assay

Viability of HEp-2 cells after exposure to varying doses of minocycline was determined using the MTT assay (Promega, CellTiter 96^®^ Non-Radioactive Cell Proliferation Assay). Briefly, in 96-well plates, 2 × 10^4^ cells/well were grown for 24 h (at 37 °C, 5% CO_2_) in MEM containing PKS and 10% FBS, next day cells were washed with Hanks’s balanced salt solution (HBSS), minocycline was added (5–40 µg/mL) with MEM containing PKS and 2% FBS, and cells were incubated for 48 h. MTT dye (15 µl) was added and incubated for 4 h. Formazan formation was stopped with 100 µL stop solution and read at 572 nm.

### 2.3. Assessing Minocycline against RSV

Based on the MTT results, the minocycline dose was selected for RSV inhibition studies by the plaque reduction assay. The procedure involved plating 2 × 10^5^ HEp-2 cells/well in a 12-well plate followed by incubation until cells were 80–90% confluent. Cells were then washed with HBSS and primed with minocycline (5, 10, 20, 40 µg/well) in 100 µl MEM (no FBS) for 1 h, followed by RSV infection (100 PFU/well) for 1 h or vice versa (infect with RSV for 1 h, followed by minocycline). The plate was rocked every 15 min during both the minocycline and RSV attachment incubation periods. Plaquing media, which is methyl cellulose-Dulbecco’s modified eagle medium (PKS and 2% FBS), was laid on the cell monolayer to make up the volume to 1 mL and cells were observed daily for development of a cytopathic effect. Cells were fixed with ice-cold methanol after 4–5 days of incubation and the cell monolayer was stained using 0.1% crystal violet to observe the plaques. The observed plaques were counted for minocycline treated cells and compared with the RSV infected (untreated) cells.

With the same experimental setup, based on the plaque reduction assay the efficacy was visualized at a sub-lethal concentration of minocycline (10 µg/mL), except that MEM containing 2% FBS (and no methyl cellulose-overlay) was used instead of plaquing media. Cells were imaged under bright field with a Nikon Ti-Eclipse microscope (at 100× magnification) after 48 h of RSV infection. These cells were later trypsinized and cell viability was determined using trypan blue staining.

### 2.4. Time-Dependent RSV Inhibition

HEp-2 cells (2 × 10^5^/well) were plated in a 12-well plate and incubated for 24 h and then washed with HBSS before adding 100 µl minocycline (10 µg/well) to the cells. After 1 h, 100 plaque-forming units (PFU)/well of RSV were added and incubated for 1 h, and plates were rocked at 15 min intervals. Finally, the total volume was adjusted to 1 mL with MEM (2% FBS) and plates were incubated at 37 °C for various time-points. The media and cells were collected after a total incubation of 2, 6, 12, or 24 h post RSV infection.

### 2.5. cDNA Synthesis and qPCR

RNA was extracted from the cells described in Section 2.4 at specific time-points using the RNeasy Mini Kit, and cDNA was synthesized with oligo-dT primers using SuperScript^®^ II Reverse Transcriptase (Invitrogen™, Carlsbad, CA, USA) as instructed by the manufacturer. Absolute quantification of RSV F gene copies was performed after the minocycline treatment in a time-course manner by real-time PCR using TaqMan Fast Advanced Master Mix (Thermo Scientific, Applied Biosystems, Waltham, MA, USA), forward primer -5′AACAGATGTAAGCAGCTCCGTTATC 3′, reverse primer -5′CGATTTTTATTGGATGCTGTACATTT 3′, and probe -5′ TGCCATAGCATGACACAATGGCTCCT 3′ as previously described [14,15]). Caspase (CASP-1) gene expression was determined (for 24 h time point) using primer-TaqMan probe set (Hs00354836_m1 Thermo Scientific, Waltham, MA, USA). RT-qPCR was performed with fast chemistry and conditions using the Applied Biosystem ViiA 7 real time PCR instrument (Thermo Scientific, Waltham, MA, USA). The GAPDH house-keeping gene was used to calculate ΔCt, and gene regulation was normalized with the untreated HEp-2 cells for calculating ΔΔCt.

### 2.6. Cytokine Response

HEp-2 cells were infected with RSV as described above in Section 2.4 and supernatants were collected at 24 h of minocycline treatment and RSV infection. The supernatants were centrifuged at 14,000 × g for 10 min to get rid of cellular debris and used for quantification of IL-6, IL-8, CXCL-10, IL-12p40 and TNF-α by specific ELISAs (BD-Biosciences, San Jose, CA, USA).

## 3. Results

### 3.1. Minocycline Reduces the Cytopathic Effect Induced by RSV

**Figure 1:**
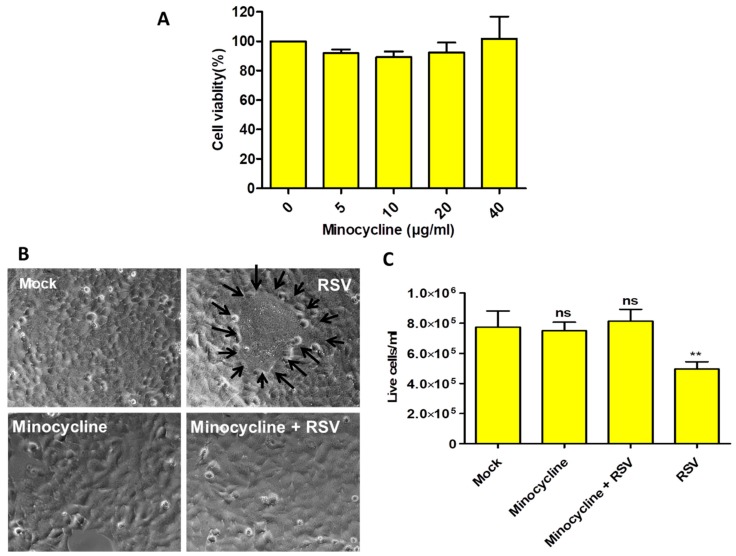
(**A**) The MTT assay showed that minocycline treatment on HEp-2 cells does not cause cytotoxicity. (**B**) Representative images of HEp-2 cells after 48 h of minocycline (10 µg/mL) or/and RSV, cells treated with minocycline and RSV and RSV infected cells (black arrows surround the syncytia; 400× magnification). (**C**) The corresponding live cell counts for the treatment groups by trypan blue staining. *n* = 3, values in the graphs represent mean with standard deviation (as error bar) with One-way ANOVA, Tukey’s multiple comparison test, significance level * *p* ≤ 0.05 or ** *p* ≤ 0.01 or *** *p* ≤ 0.0001 or ns as non-significant.

The optimal concentration of minocycline was determined by performing the MTT assay on HEp-2 cells, and the effect of various minocycline concentrations (5, 10, 20, and 40 µg/mL) was tested on cell viability after 48 h. Our results showed that minocycline had no significant effect on cell viability at these concentrations (Figure 1A).

Microscopic examination showed that HEp-2 cells (mock) and minocycline-treated cells appeared similar in their overall morphology. However, cells infected with RSV had the classical syncytia formation (Figure 1B) and cell death. The minocycline primed RSV-infected cells showed no noticeable difference when compared with uninfected cells (Figure 1B). These treatments were assessed with trypan blue staining, which indicated that minocycline protected the cells from the RSV-mediated cytopathic effect (Figure 1C). There was no significant difference in the live cell counts among the mock, minocycline primed (uninfected) and RSV infected-minocycline primed cells. Only the RSV infected cells that did not receive minocycline showed significant cell death. Thus, our data show that minocycline priming protects the cells from RSV infection.

### 3.2. Minocycline Inhibits RSV Infection

**Figure 2:**
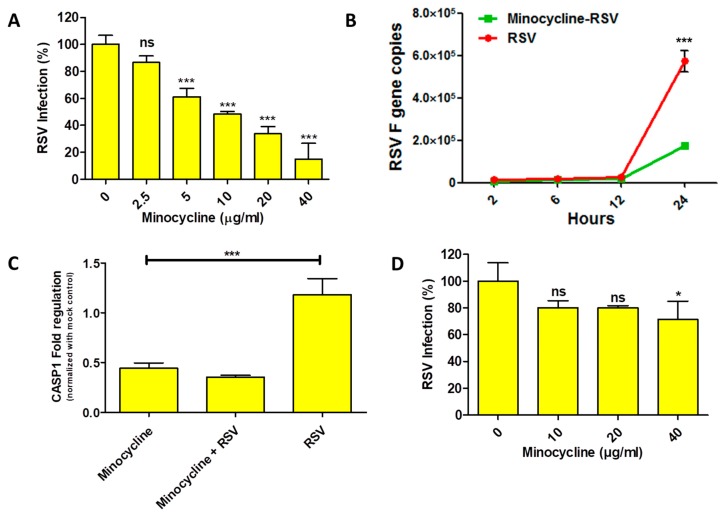
(**A**) Plaque reduction assay showing RSV inhibition by minocycline is represented as percentage of reduction in RSV infection; transformation derived from RSV counts (plaque forming units) (One-way ANOVA, Tukey’s multiple comparison test). (**B**) RSV F gene quantification for RSV infected cells that were minocycline treated at 10 µg/mL (green squares) and untreated cells (red circles) (Two-way ANOVA, Bonferroni post-test). (**C**) CASP1 gene regulation (after 24 h of treatment) normalized with GAPDH for minocycline, minocycline + RSV and RSV infected cells compared to mock-treated control. (**D**) Plaque reduction assay when RSV was allowed to infect for 1 h followed by minocycline treatment, the inhibition was statistically significant at 40 µg/mL. *n* = 3, values in the graphs represent mean with standard deviation (as error bar) with One-way ANOVA, Tukey’s multiple comparison test, significance level * *p* ≤ 0.05 or ** *p* ≤ 0.01 or *** *p* ≤ 0.0001 or ns as non-significant).

Our preliminary results indicated that minocycline treatment reduced the cytopathic effect due to RSV infection. To investigate antiviral activity of minocycline against RSV, we performed plaque reduction assay and real-time PCR to quantify the RSV inhibition. The minocycline priming showed dose-dependent RSV inhibition response with the plaque reduction assay (Figure 2A). RSV inhibition was significant (only 60% infection) at a minimum minocycline concentration of 5 µg/mL, which was further dramatically reduced to ~10% at 40 µg/mL. Based on the dose response, the IC_50_ and IC_99.9_ values (R^2^ = 0.9459, best fit value) for minocycline inhibiting RSV were found to be 6.835 µg and 10.13 mg, respectively. To get an idea of the early events of minocycline-mediated RSV inhibition, we performed real-time PCR to enumerate the RSV F gene copies as a measure of RSV infection. In the RSV infected cells, we observed a steady increase in the RSV F copies until 12 h, followed by a sudden increase at 24 h. Although a similar trend was evident with the minocycline-treated RSV infected cells, however, the copy numbers of RSV F gene were lower than the RSV-infected cells control (Figure 2B). As we learned from trypan blue assay (Figure 1C) that minocycline treatment protects from RSV induced cell death; we wanted to assess the effect of minocycline (which inhibits apoptosis) on caspase-1 (initiates apoptosis) gene expression with respect to RSV. It was interesting to see that the caspase-1 gene expression was reduced after minocycline treatment. There was a 0.82-fold reduction in caspase-1 expression in minocycline treated cells that were RSV infected (Figure 2C). This clearly shows that RSV inhibition is achieved with reduced apoptosis after minocycline treatment in the RSV infected cells. However, when cells were infected with RSV and then treated with the minocycline, the inhibition of RSV was drastically lowered (compared to minocycline primed cells followed by RSV infection). The inhibition of RSV was observed only at a dose of 40 µg/mL (Figure 2D).

### 3.3. Effect of Minocycline on Cytokines

**Figure 3:**
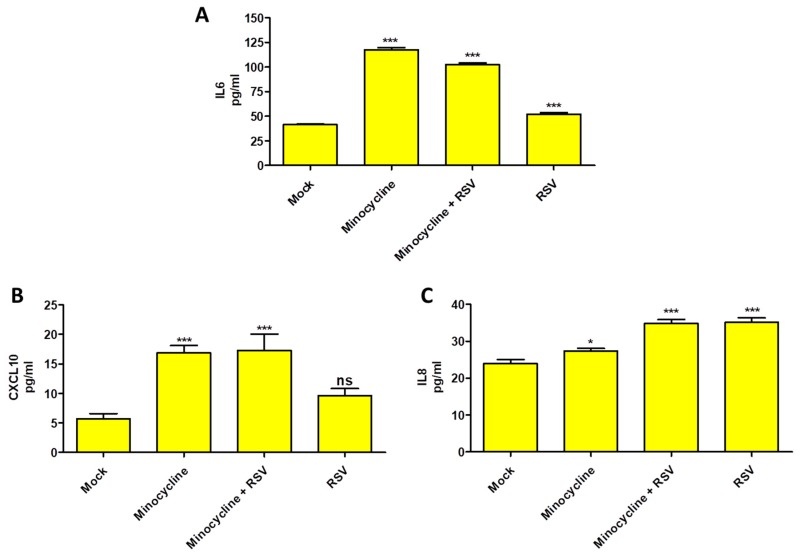
Cytokine production at 24 h in minocycline, minocycline + RSV and RSV treatment groups compared to mock control by ELISA (**A**) IL-6, (**B**) CXCL10, (**C**) IL8. *n* = 3, values in the graphs represent mean with standard deviation (as error bar) with One-way ANOVA, Tukey’s multiple comparison test, significance level * *p* ≤ 0.05 or ** *p* ≤ 0.01 or *** *p* ≤ 0.0001 or ns as non-significant.

Having demonstrated the efficacy of minocycline to reduce RSV infection and cellular cytopathic effects, we explored the effect of minocycline on cytokines production. This is important as RSV is known to modulate cytokine production during infection, especially by NS1 and NS2 proteins. We observed an increased IL-6 and CXCL10 (IP-10) production in minocycline treated cells with or without RSV infection. However, that was not the case with the IL8 production, although the levels were lower in minocycline treated (uninfected) compared to mock control (Figure 3). We did not observe detectable IL-12p40 and TNF-a production in any of the treatment groups (data not shown).

## 4. Discussion

There are diverse opinions regarding the efficacy of ribavirin, creating a scope for alternative treatment drugs against RSV. Although the outcome of ribavirin treatment for RSV is not very promising, it still remains the only drug of choice. The time required for drug development and the associated challenges are a major hurdle in RSV therapeutics. However, discovering alternative usage of already approved drugs for potential therapeutics can be possible using available computational and bioinformatics tools. Such a study was conducted by Smith et al. [16], in which they investigated the gene expression dataset to identify the drug targets for multiple respiratory viruses. Computational modeling and docking studies have proposed that doxycycline and minocycline bind Crimean-Congo virus nucleoprotein [17].

Antiviral activity of minocycline has been demonstrated by different research groups with JEV, HIV, SIV, WNV, and influenza virus. Recently, minocycline has been shown to reduce dengue virus (DV) infection [18]. Our study shows that minocycline not only reduces the RSV-mediated cytopathic effect, but also inhibits RSV. It is a possibility that minocycline affects F protein production or maturation process since we observed a reduction of RSV F gene copies, and the efficacy of RSV inhibition by minocycline is reduced if the cells are infected first and then treated with minocycline. However, there is no available literature to support this hypothesis and our findings do not exclusively suggest this mechanism of anti-RSV activity.

Minocycline did not cause any adverse effect on HEp-2 cells even at 40 µg/mL concentration. The cytopathic effect caused by RSV infection was reduced in minocycline treated cells and there was no significant cell death (Figure 1). The CASP1 gene expression result supports this observation (Figure 2C). The IL-6 and CXCL10 levels were elevated in the minocycline (non-infected or infected) treatment (Figure 3). Minocycline is known to have an anti-inflammatory effect, and we suspect it is somehow mediated by IL-6. Interestingly, IL-6, although a pro-inflammatory cytokine, can regulate innate immune response and can have an anti-inflammatory effect [19,20]. The RSV NS1 and NS2 proteins inhibit IFN-α and IFN-β [21] to establish infection, however, in our study we observed an increase in CXCL10, an interferon induced protein, after minocycline treatment. This is indicative of increased innate immune response mounted by minocycline and the fact that we showed RSV inhibition. In the case of WNV ex vivo spinal cord slice culture model, IL-6 and CXCL10 were increased upon minocycline treatment [22]. There are multiple factors that can be attributed to the inhibition of RSV by minocycline, which needs further studies.

It should be noted that our study is an explorative and observational account of antiviral activity of minocycline against RSV. Minocycline is shown to be neuroprotective [23,24] in PD [25] and Huntington’s disease models [26] and also inhibit viruses that infect (JEV and WNV) or have potential to progress into the brain (DV and HIV). Therefore, we attempt to suggest and discuss possible insights related to RSV and its association with the nervous system that may explain the efficacy of minocycline against RSV. The presence of RSV genome [27,28,29] and anti-RSV antibodies [30] in cerebrospinal fluid (CSF) in children, and/or abnormal CSF cytokine levels in children infected with RSV, clearly suggest some interplay between RSV and the nervous system [28,29,31]. Previous studies have indicated that Parkin-Ubiquitin Proteasomal System (Parkin-UPS) pathway is activated in the host upon RSV infection [16,32]. This pathway is evident in the progression of neurodegenerative Parkinson’s disease (PD) [33], but activation of this pathway in RSV infection seems interesting. There is no clear established role of RSV in neurodegenerative diseases, but it is interesting to know that RSV infection caused learning impairment in mice [34]. There are number of clinical cases that suggest association of RSV infection with neurological complications or encephalopathy [35,36,37,38]. It has been shown that RSV is capable of infecting the neuronal cell line, Neuro2a [39], primary neurons, and the lungs processes [40], indicating a link between the (lung) immune response and neuronal cells [41]. In our study, we observed that minocycline prevents RSV infection, which may imply that minocycline may be acting on some common targets that are involved during RSV infection and in some neurodegenerative disease like PD. It is not clear how minocycline prevents RSV infection, however, the reduced cytopathic effect in the minocycline-treated cells may be explained based on the downregulation of CASP1 (caspase-1) in these cells. Minocycline is known to inhibit apoptosis and down regulate CASP1 [42], but the role of CASP1 in Parkin-UPS pathway or anti-viral activity is not clear [32].

Modulation of immune response is one of the interesting properties of minocycline that we tried to address in our study. Overall, minocycline treatment increased the CXCL-10, IL-6, and IL-8 in the HEp-2 cells irrespective of RSV infection, but we did not observe detectable IL-12p40 and TNF-α production. We suggest that the increase in CXCL-10, IL-6, and IL-8 production may be responsible for RSV inhibition, which may be relevant considering that the RSV NSI and NS2 proteins manipulate host interferon responses [43]. Minocycline exhibits an anti-inflammatory effect in the case of WNV [22]; however, in this case, we find cytokine production to be pro-inflammatory with the exception of IL-6. It is difficult to comprehend the exact cytokine interplay and deduce a conclusion for RSV.

## 5. Conclusions

Reviewing the host–pathogen gene expression profiles gives insight for screening the putative targets against pre-existing drugs. Extracting information from high-throughput data and testing the inference seems a judicious strategy to identify potential drugs for RSV treatment. Our study shows that minocycline reduces RSV-mediated cytopathic effects and can prevent RSV infection. However, to understand the antiviral mechanism of minocycline, it is important to develop a model that establishes the clear link between RSV and its neuronal impact.
